# Medication and psychotherapy in eating disorders: is there a gap between research and practice?

**DOI:** 10.1186/s40337-015-0080-0

**Published:** 2015-12-01

**Authors:** Myra Cooper, Hannah Kelland

**Affiliations:** Isis Education Centre, University of Oxford, Warneford Hospital, Oxford, OX3 7JX UK

**Keywords:** Eating disorders, Psychotherapy, Medication

## Abstract

**Background:**

Little research has investigated the use of evidence-based guidelines by eating disorder (ED) therapists, or prescribing of psychotropic medication. Moreover, people with EDs have rarely been surveyed on these topics, and their clinical and demographic features have not been presented. This study investigated perception of psychotherapy, psychotropic medication and the clinical characteristics of a community sample of people with EDs.

**Method:**

An online survey methodology was used to recruit 253 people with eating disorders in the community. Where feasible, comparisons were made between four types of eating disorder, anorexia nervosa, bulimia nervosa, and two types of atypical or ‘sub-threshold’ eating disorder.

**Results:**

Unlike medication, reported psychotherapy showed some congruence with evidence based and other guidance. Most participants were currently receiving either psychotherapy, medication or both, and most had a severe and chronic ED.

**Conclusions:**

Findings are considered in light of use of evidence-based treatment for EDs, calls for greater dissemination of cognitive behaviour therapy (CBT); indications that much may be poor quality; and the importance of what treatments to offer those who are chronically and severely ill. Development of theory and novel treatments is considered a priority.

## Background

Eating disorders (EDs) are associated with significant mortality and morbidity [1]. They can be difficult to treat, and there is room for improvement [2]. Not all community cases receive treatment [3], and not all receive “evidence-based” treatment [4]. Practice guidelines [5–8] identify cognitive behaviour therapy (CBT), and fluoxetine as the best evidence based treatments for bulimia nervosa (BN). CBT is recommended for adult anorexia nervosa (AN), “based on strong consensus but weak evidence”([6], p4). Interpersonal psychotherapy (IPT), cognitive analytic therapy (CAT) and psychodynamic therapies may be useful for AN but ‘evidence for each of these is modest’ ([8], p14). Guidelines recommend sub-threshold EDs be treated in line with the disorder they most resemble [5], although an evidence base is lacking. No drugs are specifically recommended for AN. Use of a range of psychotropic medication in is discussed in AN [5, 6, 8], but guidelines urge extreme caution, and highlight potentially serious hazards.

Few studies have investigated what treatment people with EDs in the community receive, and whether or not it meets practice guidelines. Most survey therapists not patients. Therapists report high use of psychotherapy, particularly CBT. One study [9] found 196 (74.5 %) of ED therapists employed CBT for AN, and 181 (83.8 %) for BN. However, among community clinicians there was a wider range of approaches. Few used evidence-based treatment [10], and less than a quarter, 27 (22.9 %), used CBT. When those with EDs in the community replied to a research notice, 220 (62.4 %) with probable BN reported receiving psychotherapy, although few (N = 23, 6.7 %) had received narrowly defined CBT [11]. Psychotherapy received by those with AN or sub-threshold EDs has not been studied from the patient’s perspective. Moreover, it has not always been clear whether therapy was designed to treat the ED. This is important as EDs have been associated with high co-morbidity [3].

In relation to psychotropic medication, chart review found ED patients had often received antidepressants, N = 102 (50 %), and anxiolytics, N = 32 (16 %), in the preceding 12 months, although no details of the drugs, ED or other diagnosis being treated, was provided [12]. A study of patients with AN found 278 (53 %) reported current use of psychotropic medication (N = 254, 48.4 %, antidepressants and N = 68, 13 %, antipsychotics) [13]. Exact figures are not provided, but a wide range of drugs was being taken. In the only study to report on medication and psychotherapy, 225 (63.7 %) with probable BN had received pharmacological treatment (vs N = 220, 62.4 %, psychotherapy), and 129 (36.6 %) were judged to have had an adequate course of fluoxetine [11]. As in other studies, sub-threshold EDs were not studied, no detail was provided on the drugs taken and whether the ED, or a different problem, was the target.

Importantly, no study has reported demographic and clinical information, including data collected using standardised self-report measures. This would establish a baseline against which to compare future studies as well as aid interpretation of findings. The current study therefore investigated medication and type of psychotherapy perceived to have been received by those with an ED, including sub-threshold diagnoses. Participants were questioned about treatment specifically for their ED. Information on demographic characteristics, including height and weight, and psychiatric symptoms, using standardised measures, was collected.

## Methods

### Participants

A community sample (aged 18-65) was recruited through internet advertisements on websites such as Facebook and Beating Eating Disorders (a UK charity). The text noted “we are looking for women and men who are currently suffering from an eating disorder (anorexia nervosa, bulimia nervosa and eating disorder not otherwise specified). To take part you must be 18–65 years of age and speak fluent English”.

### Design

The study was an internet-based self-report survey.

### Procedure

Approval was obtained from the University ethics board. Participants were provided with a link to the online survey. An option of completing paper copies of the questionnaires was available. All participants gave informed consent.

### Measures

Participants were asked their height and weight to calculate current BMI. They were asked details of age, ethnicity, and occupation and any psychiatric diagnoses, apart from an ED. Participants completed the following self-report questionnaires.

#### Eating disorder diagnostic scale (EDDS; 14)

This brief scale diagnoses EDs. The items map onto the Diagnostic and Statistical Manual, fifth edition (DSM-V) criteria [15] for AN and BN, and permit diagnosis of sub-threshold AN and BN (i.e. where full criteria are not met). The EDDS possesses good test-retest reliability (r = .87) and internal consistency (alpha = .89) [14].

#### Hospital anxiety and depression scale (HADS;^16^)

This questionnaire contains seven statements related to anxiety and seven related to depression. A score of 11 or more indicates probable mood disorder. The sub-scales are independent, and have good homogeneity and reliability [17].

#### Eating attitudes test (EAT; 18)

This measure of eating disturbance is an abbreviated (26-item) version of the 40-item EAT [19], and the two are highly correlated (r = .98; 18). Scores above 20 indicate potential eating disturbance. It has good reliability and validity [18].

#### Eating disorder examination questionnaire (EDE-Q; 20)

This is a self-report version of the Eating Disorder Examination [21]. It has 4 subscales, and concerns behaviour over the past 28 days. A global score can be computed. A review indicates good reliability, and supports its use in identifying cases [22].

### Medication and psychotherapy use

#### Medication use

Participants were asked whether or not they were currently taking medication in relation to their ED. They were asked to list all these.

#### Psychotherapy use

Participants were asked whether or not they had received or were currently receiving psychotherapy for their ED. They were asked to list the type of therapy/therapies.

## Results

### Data analysis

Demographic data were analysed by diagnostic group using one way analysis of variance for continuous variables and chi square for categorical data. Chi square was used to examine group differences in total number receiving psychotropic medication and current and past psychotherapy. Further statistical testing was not undertaken due to small numbers in most categories.

### Response

Four hundred and fifty four people started, and 260 (57 %) completed, the survey. Two hundred and fifty three (97.3 %) were diagnosed with AN, BN, or sub-threshold ED using the EDDS.

### Diagnosis

Extracting data from the EDDS, and using the algorithm [13], forty-four participants had a DSM-V [15] diagnosis of AN; 30 a diagnosis of atypical (sub-threshold) AN, 126 a diagnosis of BN, and 53 a diagnosis of BN of low frequency and/or duration (sub-threshold BN).

### Demographic results

Two hundred and forty six participants were female and seven were male (one AN, three BN and three sub-threshold BN). Demographic details (continuous variables) for each diagnostic group are in Table 1 and (categorical variables) in Table 2. There was no significant difference between the four groups in age (F = 1.53, df = 3, 248, *p* = .21). Most participants were in their late 20s. There was a significant difference between groups in BMI (F = 13.3, df = 3, 250, *p* < .001). Post hoc Tukey tests indicated those with AN had a lower mean BMI than all other groups (all *p* values < .03). The distribution of Caucasian/non Caucasian participants did not differ (chi^2^ = 1.62 df = 3, *p* = .65), and most were Caucasian. The majority lived in the United Kingdom or USA. Socioeconomic Status (SES) was classified using the Office of National Statistics Standard Occupational Classification [23]. Extra categories were added for students, the unemployed and retired. The majority were students although a significant number were managers/professionals or unemployed. There were no significant differences in occupation in the four groups (chi^2^ = 6.62, df = 9, *p* = .67). Over a third had been a psychiatric inpatient, particularly those with AN and sub-threshold AN (chi^2^ = 13.9, df = 3, *p* = .003). Mean duration of ED was similar across the sample (11.3 years, SD = 9.37), (F = 1.64, df = 3, 249, *p* = .18). Twenty four (11.3 %) reported a current psychiatric diagnosis other than an ED, with no differences between the groups (chi^2^ = 3.96, df = 3, *p* = .27).

**Table 1:** Demographic and clinical characteristics of the sample – continuous variables

	AN		AN-sub-threshold		BN		BN-sub-threshold		Total	
	(N = 44)		(N = 30)		(N = 126)		(N = 53)		(N = 253)	
	Mean	SD	Mean	SD	Mean	SD	Mean	SD	Mean	SD
Age	28.0	10.3	26.3	9.4	26.1	8.5	29.4	12.3	27.1	9.8
Body Mass Index	15.8	1.2	20.2	6.1	23.1	8.1	22.8	6.8	21.4	7.3
EAT	49.4	13.3	46.8	14.7	42.6	14.0	33.5	18.4	42.4	15.8
EDE-Q	4.6	1.1	4.8	0.9	4.8	0.9	3.7	1.6	4.6	1.2
HADS-depression	11.0	4.5	10.6	4.3	10.0	4.6	8.9	4.9	10.0	4.6
HADS-anxiety	15.1	4.4	14.6	3.6	14.0	3.9	13.3	4.8	14.1	4.2
Duration of ED (years)	13.3	9.4	9.3	7.5	10.6	9.0	12.5	9.4	11.3	9.4

Key: *AN* Anorexia Nervosa, AN-sub-threshold, *BN* Bulimia Nervosa, BN-sub-threshold, *EAT* Eating Attitudes Test, *EDE-Q* Eating Disorder Examination – Questionnaire, *HADS* Hospital Anxiety and Depression Scale

**Table 2:** Demographic and clinical characteristic of the sample – categorical variables

	AN		AN-sub-threshold		BN		BN-sub-threshold		Total	
	(N = 44)		(N = 30)		(N = 126)		(N = 53)		(N = 253)	
	N	%	N	%	N	%	N	%	N	%
Ethnicity										
Caucasian	41	93.2	29	96.7	114	90.5	46	86.8	230	90.9
Non Caucasian	3	6.8	1	2.3	12	9.5	3	5.7	19	7.5
Country										
United Kingdom	21	47.8	11	36.7	47	37.5	27	50.9	91	36.0
USA	12	27.3	15	50.0	44	35.0	16	30.2	78	30.8
Canada, Australia, New Zealand	8	18.2	4	13.3	18	14.3	3	5.7	31	12.3
SES										
Managers/professionals	8	18.2	4	13.3	20	15.9	6	11.3	38	15.0
Associate professionals	3	6.8	2	6.7	5	4.0	6	11.3	16	6.3
Students	14	31.8	15	50.0	44	35.0	22	41.5	95	37.5
Unemployed	6	13.6	6	20.0	23	18.3	6	11.3	41	16.2
ED/psychiatric variables										
Been a psychiatric inpatient	26	59.0	14	46.7	37	29.4	16	30.2	93	36.8
Other psychiatric diagnosis										
Depression	3	6.8	1	2.3	7	5.6	2	3.8	13	5.1
Anxiety	3	6.8	0	0	2	1.6	0	0	5	2.0
Borderline personality disorder	2	4.5	0	0	4	3.2	0	0	6	2.4
Bipolar disorder	1	2.3	1	2.3	4	3.2	0	0	6	2.4
PTSD	3	6.8	0	0	3	2.4	1	1.9	7	2.8
OCD	0	0	0	0	3	2.4	0	0	3	1.2
Self-harm	0	0	0	0	1	0.8	0	0	1	0.4
ADHD	0	0	0	0	0	0	0	0	1	0.4

Key: *AN* Anorexia Nervosa, AN-sub-threshold, *BN* Bulimia Nervosa, BN-sub-threshold *SSRIs* Selective

**Table 3:** Summary of medication taken by sample

	AN		AN-sub-threshold		BN		BN-sub-threshold		Total	
	(N = 44)		(N = 30)		(N = 126)		(N = 53)		(N = 253)	
	N	%	N	%	N	%	N	%	N	%
No medication	17	38.6	14	46.7	77	69.1	31	58.5	139	54.9
One psychiatric drug	14	31.8	9	30	22	17.5	8	15.1	53	20.9
Two psychiatric drugs	5	11.4	3	10.0	13	10.3	5	9.4	26	10.3
Three psychiatric drugs	3	6.8	4	13.3	7	5.6	3	5.7	17	6.7
Four psychiatric drugs	4	9.1	0	0	2	1.6	2	3.8	8	3.2
Five psychiatric drugs	0	0	0	0	3	2.4	0	0	3	1.2
Total on any psychiatric medication	26	59.0	16	53.3	41	32.5	25	47.2	108	42.7
Anti-anxiety	7	15.9	5	16.7	12	9.5	6	11.3	36	14.2
Hypnotics	0	0	0	0	4	3.2	2	3.8	6	2.4
Psycho-stimulants	2	4.5	0	0	3	2.4	2	3.8	7	2.8
Tricyclic antidepressants	1	2.3	0	0	1	0.8	1	1.9	3	1.2
SSRIs	20	45.5	11	36.7	8	14.3	11	20.8	50	19.8
Fluoxetine	4	9.1	5	16.7	4	3.2	2	3.8	15	5.9
SARIs	2	4.5	0	0	3	2.4	0	0	5	2.0
SNRIs	1	2.3	2	6.7	15	11.9	3	3.8	21	8.3
NaSSRAs	1	2.3	1	3.3	4	3.2	1	1.9	7	2.8
NDRIs	1	2.3	0	0	5	4.0	0	0	6	2.4
Melatonergic agonists	0	0	0	0	1	0.8	0	0	1	0.4
Antidepressant (unspecified)	1	2.3	0	0	0	0	1	1.9	2	0.8
Total on anti-depressants	27	61.4	14	46.7	41	32.5	25	47.2	107	42.3
Mood stabilisers	3	6.8	4	13.3	16	12.7	3	3.8	26	10.3
Antipsychotics	7	15.9	2	6.7	8	6.3	4	7.5	21	8.3

Key: *AN* Anorexia Nervosa, AN-sub-threshold, *BN* Bulimia Nervosa, BN-sub-threshold, *SSRIs* Selective Serotonin Reuptake Inhibitors, *SARIs* Serotonin Antagonist and Reuptake Inhibitor, *SNRIs* Serotonin Norepinephrine Reuptake Inhibitor, *NaSSAs* Noradrenergic and Specific Serotinergic Antidepressant, *NDRIs* Norepinephrine Dopamine Reuptake Inhibitor

**Table 4:** Summary of psychotherapy received by sample

	AN		AN-sub-threshold		BN		BN-sub-threshold		Total	
	(N = 44)		(N = 30)		(N = 126)		(N = 53)		(N = 253)	
	N	%	N	%	N	%	N	%	N	%
Current therapy	27	61.4	16	53.3	61	48.4	19	35.8	123	48.6
CBT	14	31.8	10	33.3	25	19.8	10	18.9	110	23.3
Schema focussed therapy	1	2.3	0	0	0	0	0		1	0.4
Family therapy	6	13.6	3	10.0	5	4.0	2	3.8	16	6.3
Psychodynamic therapy	2	4.5	2	6.7	7	5.6	2	3.8	13	5.1
CAT	1	2.3	0	0	1	0.8	0	0	2	0.8
DBT	1	2.3	2	6.7	6	4.8	0	0	9	3.6
Counselling	1	4.5	2	6.7	3	2.4	2	3.8	8	3.2
Other	7	15.9	5	16.7	10	7.9	3	5.7	25	9.9
Don’t know type of therapy	5	11.4	6	20.0	21	16.7	12	22.4	44	17.4
Past therapy	41	93	25	83.3	102	81	39	73.6	207	81.8
CBT	23	52.3	19	63.3	52	41.3	25	47.2	119	47.0
Schema focussed therapy	1	2.3	2	6.7	4	3.2	1	1.9	8	3.2
Family therapy	14	31.8	12	40.0	22	17.5	12	22.4	60	23.7
Psychodynamic therapy	11	25.0	4	13.3	18	14.3	10	18.9	43	17.0
CAT	3	6.8	1	3.3	4	3.2	5	9.4	13	5.1
DBT	2	4.5	0	0	2	1.6	1	1.9	3	1.2
Counselling	2	4.5	2	6.7	2	1.6	1	1.9	7	2.8
Other	3	6.8	1	3.3	9	7.1	4	7.5	17	6.7
Don’t know type of therapy	8	18.2	5	16.7	38	30.2	10	18.9	61	24.1
Current and past psychological therapy	27	61.4	13	43.3	53	42.1	17	32.1	110	43.5
Combination therapy	19	43.2	11	36.7	36	28.6	9	17.0	75	29.6
Psychotherapy only	27	61.4	5	16.7	23	18.2	10	18.9	56	39.5
Medication only	7	15.9	5	16.7	13	10.3	9	17.0	30	11.9
No therapy	8	18.2	10	33.3	47	37.4	7	13.2	72	28.5

Key: *AN* Anorexia Nervosa, AN-sub-threshold, *BN* Bulimia Nervosa, BN-sub-threshold, *CBT* Cognitive Behaviour Therapy, *CAT* Cognitive Analytic Therapy, *DBT* Dialectical Behaviour Therapy

### Self-report questionnaires

Scores for the diagnostic groups on the questionnaires are in Table 1. There was a significant difference between the groups in EAT scores (F = 10.29, df = 3252, *p* = <.001). Post hoc Tukey tests found the sub-threshold BN group had a significantly lower score than the other groups (all *p* values < .002). There was a significant difference between groups in EDE-Q total score (F = 13.76, df = 3, 252, *p* < .001). Post hoc Tukey tests indicated a lower score in the sub-threshold BN group compared to all other groups (all *p* values < =.001). There was no significant difference between the four groups in HADS depression (F = 1.8, df = 3252, *p* = .15) or anxiety (F = 1.59, df = 3, 252, *p* = .19).

### Medication use

Table 3 displays psychotropic medication use for the four diagnostic groups and whole sample. There was a significant difference between the groups (chi^2^ = 9.62, df = 3, *p* = .02) in whether or not any psychotropic medication was being taken. It was most common in AN (N = 26, 59 %), and least common in BN (N = 41, 32.5 %). Overall, a substantial minority (N = 54, 10.2 %) were taking two or more psychotropic drugs. Antidepressants were the most common (taken by N = 107, 42.3 %), with N = 50, 19.8 % on Selective Serotonin Reuptake Inhibitors and N = 21, 8.3 %, on Selective Norepinephrine Reuptake Inhibitors. A minority were taking fluoxetine (N = 15, 5.9 %); four (3.2 %) had BN and two (3.8 %) had sub-threshold BN. A significant minority were taking anti-anxiety drugs (N = 36, 14.2 %), mood stabilisers (N = 26, 10.3 %) and antipsychotics (N = 21, 8.3 %).

### Psychotherapy use

Table 4 displays current and past psychotherapy use for the whole sample and each ED diagnosis. Just under half were receiving current psychotherapy (N = 123, 48.6 %), with no group differences (chi^2^ = 4.97, df = 3, *p* = .17). CBT was being received by N = 110, 23.3 % of the total sample, and family therapy by a small number of those with AN (N = 6, 13.6 %) and sub-threshold AN (N = 3, 10 %). Overall, few were receiving Cognitive Analytic Therapy (CAT) (N = 2, 0.8 %), Dialectical Behaviour Therapy (DBT) (N = 9, 3.6 %), Interpersonal Psychotherapy (IPT) or Counselling (N = 8, 3.2 %). A number did not know what type of psychotherapy they were receiving (N = 44, 17.4 %). Some were receiving a range of “other” forms of therapy (N = 25, 9.9 %).

81.8 % (N = 207) had received past psychotherapy, with no group differences (chi^2^ = 4.0, *p* = .26). Just under half had received CBT (N = 110, 47 %). A number with AN (N = 14, 31.8 %), or sub-threshold AN (N = 12, 40 %) had received family therapy. A significant minority had received psychodynamic therapy (N = 43, 17 %), some had received CAT (N = 13, 5.1 %), DBT (N = 3, 1.2 %), IPT or Counselling (N = 7, 2.8 %). A number did not know what type of therapy they had received (N = 61, 24.1 %).

### Combination therapies

Just under half who had received past psychological therapy were currently receiving it. Nearly one third (N = 75, 29.6 %) was currently receiving both psychological therapy and psychotropic medication (highest in AN, N = 19, 43.2 %). Thirty people (11.9 %) were currently taking medication only (highest in sub-threshold BN, N = 9, 17 %). Thirty-nine people (18.3 %) were receiving psychotherapy only (highest in AN, N = 27, 61.4 %). Overall, just over one quarter were not receiving current treatment (N = 72, 28.5 %), highest in BN (N = 47, 37.3 %) and sub-threshold AN (N = 10, 33.3 %), and lowest in AN (N = 8, 18.2 %), and sub-threshold BN (N = 7, 13.2 %).

## Discussion

A community sample was asked about treatment for their ED. Just under half were taking psychotropic medicine, with few taking fluoxetine. Nearly half were receiving psychotherapy, often CBT. A wide range of psychotropic and psychological therapies was being received. Most had previously received psychotherapy. Just under a third was receiving both medication and psychotherapy.

The sample was demographically similar to clinical samples (including AN, BN and sub-threshold diagnoses in the community, 24), but with more severe psychopathology and longer illness duration. Indeed, our sample had severe and chronic EDs, and the findings should be interpreted in this light. Interestingly, those with sub-threshold diagnoses had symptom scores similar to those with AN or BN, and illness of similar duration, suggesting sub-threshold disorders can also be severe and chronic. To date, treatment received has usually been studied only in AN or BN, has not investigated medication and psychotherapy, nor reported ED symptom severity, thus enabling therapy data to be contextualised. Unlike most studies we asked patients (not clinicians) what treatment was received, gaining a view of what patients perceived (particularly important for type of psychotherapy). Most had previously received psychotherapy for their ED, including those with sub-threshold diagnoses. This is consistent with the finding that many who present to services have such diagnoses, and that these are relatively common ED diagnoses [25]. Nearly half were currently taking psychotropic medication, and just over half were receiving psychotherapy. Some (32.9 %) were receiving both. Receipt of psychotropic medication is similar to previous studies [12, 13]. However, the percent (7 %) receiving fluoxetine was markedly lower [11]. Indeed, it is surprisingly low, given fluoxetine is recommended by NICE for BN and sub-threshold BN. However, some guidelines are less enthusiastic about prescribing fluoxetine, highlighting lack of follow-up, and high attrition [8]. Overall, however, nearly half was taking an antidepressant, often an SSRI, but like previous studies, a wide range of other antidepressants [13]. A significant minority were taking anti-anxiety drugs, mood stabilisers, and antipsychotics. This is consistent with in-patients with AN [13] and extends these findings to other diagnoses and the community. However, it is important to note that none of these medications has an evidence base in EDs, and some may be contraindicated because of safety, particularly in AN [6–8, 13]. Just over half our sample was receiving CBT, and some with AN or sub-threshold AN, family therapy. This is consistent with the evidence base in BN, and recommendations for AN. However, several other psychological therapies were being received, most with minimal evidence base in EDs. Worryingly, many did not know what type of psychological therapy they were, or had been, receiving. Over half receiving psychotherapy had previously received psychotherapy, suggesting it had not resolved the ED, they had experienced a recurrence of symptoms or perhaps had not completed or engaged with treatment, all of which are known problems in EDs. These possibilities seem particularly likely given the chronicity and severity of the sample. For example, use of non-evidence based therapy currently may be due to previous failure of evidence-based treatment. One particular limitation here is that while we asked about previous psychotherapy we did not ask about previous medication; thus previous use of evidence-based medication could not be evaluated, and it may be that fluoxetine (for example) had not proved helpful for our patients in the past and thus more diverse drugs had been prescribed. Self-reported comorbidity was low, and the reason for this is unclear. One possibility is that it is less likely to be identified and labelled outside highly structured research, another that it is often considered a secondary problem.

Despite encouraging trends for CBT and family therapy, the fit between our data and ED (evidence-based) practice guidelines is not close. A plethora of different treatments (some evidence based, but many not) had been, and were being, received. Explanations for treatment failure in EDs vary from lack of dissemination of CBT to poor patient motivation/engagement [26]. While these may play a role, what is striking about the current sample is that all had a severe and chronic ED. An alternative to these different explanations for treatment failure may be that, in fact, we currently lack effective, powerful treatments for EDs. The question is not, why don’t more people receive CBT or fluoxetine? It may not be why is better quality treatment not available? The question may be why is there not more emphasis on the development of theory to inform novel treatment approaches? For some with a chronic and severe ED the range of treatments tried (understandably given failure to recover) is truly staggering.

The study has limitations. The majority of participants had received treatment; the sample is unlikely therefore to represent all those with EDs in the community (27). Diagnosis was completed using self-report questionnaire, likely less valid than clinician obtained diagnosis. The sample was relatively ill with severe symptoms and a chronic course, thus not representative of those with less severe symptoms and recent onset. Indeed, there may have been a selection bias in that the survey appealed to those with chronic and severe EDs. Self-report of medication and psychotherapy may be less reliable and valid than chart review. Data on quality, frequency, duration or dosage of the interventions, or individual adherence, was not obtained. Finally, given clinician self-report of evidence based treatment may lack validity [4], this may also be problematic for patient self-report of these.

## Conclusions

It is easy to criticise non-evidence based psychotherapy, and the apparently widespread use of psychotropic medication that lacks an evidence base for EDs. As their scores and history indicate, this sample is seriously and chronically ill. It is not easy for therapists and services to do nothing. If CBT has failed (however well delivered), it is hard to justify providing it again, and perhaps understandably, patients and therapists may seek alternative psychotherapies. It is not clear why fluoxetine is given or taken so rarely (some suggestions are given above). However, it is clear that cautious and carefully monitored use of a number of non-evidenced based psychotropic medications can, as suggested by the APA Practice Guidelines for the Treatment of Patients with EDs [6], provide some relief from troubling symptoms, even if not directly treating the ED. In the absence of any real ED specific medication this is likely to be a useful option for some, and one that it would not be appropriate to withhold from patients. The same may also be true of some non-evidence based psychotherapy. Finally, it may be mistaken to conclude that individuals in our sample have not benefitted from at least some of the interventions received. While efficacy studies focus on modifying diagnostic features, clinicians treating those who are very ill, are likely to be rather more concerned with quality of life. A wide range of therapies may be needed to achieve this, including those not specifically focussed on the ED.
